# Health-related quality of life and strain in caregivers of Australians with Parkinson’s disease: An observational study

**DOI:** 10.1186/1471-2377-12-57

**Published:** 2012-07-17

**Authors:** David H Kelly, Jennifer L McGinley, Frances E Huxham, Hylton B Menz, Jennifer J Watts, Robert Iansek, Anna T Murphy, Mary Danoudis, Brooke Adair, Meg E Morris

**Affiliations:** 1 Physiotherapy, Melbourne School of Health Sciences, The University of Melbourne, Carlton, VIC, Australia, 3010; 2 National Parkinson Foundation Center of Excellence, Clinical Research Center for Movement Disorders and Gait, and Victorian Comprehensive Parkinson’s Program, Kingston Centre, Warrigal Road, Cheltenham, VIC, Australia, 3192; 3 Musculoskeletal Research Centre, Faculty of Health Sciences, La Trobe University, Bundoora, VIC, Australia, 3086; 4 Center for Health Economics, Monash University, Building 75, Clayton, VIC, Australia, 3800

## Abstract

**Background:**

The relationship between health-related quality of life (HRQoL) in people with Parkinson’s disease and their caregivers is little understood and any effects on caregiver strain remain unclear. This paper examines these relationships in an Australian sample.

**Methods:**

Using the generic EuroQol (EQ-5D) and disease-specific Parkinson’s Disease Questionnaire-39 Item (PDQ-39), HRQoL was evaluated in a sample of 97 people with PD and their caregivers. Caregiver strain was assessed using the Modified Caregiver Strain Index. Associations were evaluated between: (i) caregiver and care-recipient HRQoL; (ii) caregiver HRQoL and caregiver strain, and; (iii) between caregiver strain and care-recipient HRQoL.

**Results:**

No statistically significant relationships were found between caregiver and care-recipient HRQoL, or between caregiver HRQoL and caregiver strain. Although this Australian sample of caregivers experienced relatively good HRQoL and moderately low strain, a significant correlation was found between HRQoL of people with PD and caregiver strain (*rho* 0.43, *p* < .001).

**Conclusion:**

Poor HRQoL in people with PD is associated with higher strain in caregivers. Therapy interventions may target problems reported as most troublesome by people with PD, with potential to reduce strain on the caregiver.

## Background

Little is known about health-related quality of life (HRQoL) in people with Parkinson’s disease (PD) and their caregivers in Australia, or of the level of strain experienced by family caregivers of people with PD. This paper investigates those issues and explores their relationships.

Informal caregivers are estimated to provide some $AUD30 billion support annually to people with disabilities in Australia alone [1]. In the USA, this figure was estimated to be as high as $US375 billion in 2007 [2] and currently £119 billion in the UK [3]. In the case of PD, family members and particularly spouses are usually the primary caregivers [4,5]. Their support often enables people with PD to remain at home despite increasing disability, significantly reducing the costs associated with a move to residential care [5,6]. Together with physical assistance, family members can provide emotional and practical support that helps maintain better quality of life in the person with PD and may reduce morbidity and mortality [7].

The vital support provided by informal caregivers is not always without cost to the carers. There is a growing body of literature detailing some of the negative sequelae of PD on some caregivers’ lives. People providing care for others with advanced PD have demonstrated an increased incidence of depression [8,9], diminished quality of life [10], reduced economic circumstances [11,12] and reduced physical [5] and mental health [5,10,13]. These and other factors brought about by stress and burden can combine to cause increased levels of caregiver strain, which has been defined as “an enduring change … in a caregiver’s fabric of well-being” [14].

With the importance of the care-giving role now recognised, research attention is turning to the interplay between caregiver and care-recipient. Understanding which aspects of the care-recipient’s condition are most likely to negatively affect caregiver wellbeing may enable therapy interventions to be targeted to assist both patient and caregiver [15], such as addressing factors contributing to decreased HRQoL.

Previous research has shown that quality of life in people with PD and their caregivers is linked [5]. Health-related quality of life refers to facets of an individual’s self-perceived well-being that are affected by disease or treatment [11], suggesting that it may respond to appropriate therapy interventions. Parkinson’s disease is known to reduce HRQoL in both people with PD [11] and caregivers in general [7], yet much less is known about the relationship between HRQoL in care-recipient with PD and caregiver, or between caregiver HRQoL and caregiver strain in those who care for individuals with PD.

In this study, we report the HRQoL of an Australian sample of people with PD and their caregivers, and examine the level of strain in caregivers. A key aim was to consider the relationship between HRQoL in people with PD and their caregivers. Further aims were to determine the associations between caregiver HRQoL and caregiver strain; and HRQoL in PD and caregiver strain.

## Methods

### Study design

The study formed part of a larger clinical randomized controlled trial that investigated the efficacy of two physiotherapy programs relative to a control intervention to reduce falls and improve mobility in people with PD (see [16] for full details). Participants were drawn from general medical practitioners, neurologists, Parkinson’s Victoria network and advertisements in local papers. As part of their baseline assessments, participants with PD were asked if they had a significant other person in their life whom they could designate as a caregiver. The current study thus utilized a cross-sectional design. The study design and content were approved by the Human Research Ethics Committee of The University of Melbourne (HREC Number 0829579.2).

### Participants

Participants with PD had to meet the criteria for the primary investigation which were: a diagnosis of idiopathic PD; Hoehn and Yahr score less than V, equating to the ability to walk independently (with or without an assistive device); Mini Mental State Examination score [17] of 24 or greater; ability to participate safely in an exercise program and to provide informed consent. Caregivers were included in the current study if they were nominated as the main caregiver by the person with PD, were willing to provide informed consent, and were able to read and complete the necessary questionnaires.

### Procedure

Information from care-recipients was collected as part of the baseline assessment protocol of the larger study [16], performed by trained physical therapists in an outpatient setting. Caregiver assessments were performed at the same time if they had accompanied their care-recipient, or by postal completion of questionnaires.

### Outcome measures

#### Health related quality of life

The EuroQoL (EQ-5D) was chosen as the common HRQoL tool for both people with PD and their caregivers [18]. It utilises five questions covering mobility, personal care, usual activities, pain / discomfort and anxiety / depression, each with three options and summing to a single score. The overall health state is subsequently defined by a five-digit number. Comparisons with healthy older people and other patient groups are available [18]. A Visual Analogue Scale (VAS) is included, depicting the subject’s perceived health on a scale of 0 (death) – 100 (perfect health) on that day. The EQ-5D is sensitive to the clinical features of the Parkinson’s Disease Questionnaire-39 (PDQ-39) [19] and correlates strongly with both PDQ-39 and Hoehn and Yahr scores [20]. Country specific weightings have been developed for the EQ-5D which reflect the respective population data of the country from which they have been derived. As no Australian weights (Wt) are available, the VAS and time trade off (TTO) weighted scores were calculated using the United Kingdom weights and formulae as described by Szende et al. [21].

The disease-specific PDQ-39 [22] was also used to evaluate HRQoL in the participants with PD. The PDQ-39 uses 39 questions with five levels of choice and covers eight domains, including mobility, activities of daily living and emotional well-being. These are weighted to provide a single score, with higher scores denoting lower HRQoL. The PDQ-39’s strong measurement qualities [11] make it the most widely used disease-specific tool in Parkinson’s disease.

#### Caregiver Strain

Caregiver strain or enduring change in wellbeing was evaluated using the Modified Caregiver Strain Index (MCSI) [23,24]. This instrument uses 13 questions scored at three levels to assess the impact of caring on the provider’s health, finances, social interactions, time demands and employment. Scores from 0 to 2 for each question lead to a maximum of 26, denoting maximal strain.

#### Other measures

Motor performance in participants with PD was assessed by the timed 6-metre walk test (6MWT), which has demonstrated reliability in people with PD [25]. The modified Hoehn and Yahr scale [26] was used to provide an overall view of the level of PD severity, where I denotes mild unilateral symptoms progressing to an inability to walk at level V.

### Statistical analysis

Descriptive data were summed and tabulated. As scoring on the EQ-5D, PDQ-39 and MCSI is ordinal, the non-parametric Spearman rank correlations coefficient (r_s_) was used to examine associations between measures. All analyses were performed using PASW Statistics 18.0 (SPSS) and IBM SPSS Statistics 19.0.

## Results

The primary RCT included 210 people with PD, with data for the current study available from 97 caregiver–recipient dyads. Seven missing data points in PDQ-39 (0.15% of data) were filled using the expectation minimization algorithm [27].

### Participant Characteristics

Participants with PD were predominantly men (64%) in their late 60s (Table 1). There was a spread of disease severity as measured by the modified Hoehn and Yahr status [26] with the largest number (54%) moderately disabled at level 3 and above. A further 32% were classified as level 2 or 2.5. Although only two percent required supported accommodation, the higher number with moderate to severe disease suggests considerable demands on some caregivers, as carer strain is known to increase with Hoehn and Yahr level [28].

**Table 1:** Characteristics of participants with PD

**Participant characteristics**	**PwPD (n = 97)**
Age (years); mean (SD)	68.5 (9.5)
Gender, n females (%)	35 (36)
Disease duration (years); mean (SD)	7.5 (5.8)
Modified Hoehn and Yahr stage; n (%)	
< 1.5	8 (8)
1.5	5 (5)
2.0	19 (20)
2.5	12 (12)
3	40 (41)
4	13 (13)
Timed 6 Metre Walk Test ^**a**^ (seconds); mean (SD)	5.8 (2.1)

^a^ Two participants with missing data.

Most caregivers (84%) were spouses/partners, with 13% being their children. Sixty-six percent of caregivers were over 60 years old, seven percent were over 80 and six percent were less than 40 years old.

### HRQoL in PwPD and their caregivers and caregiver strain

HRQoL scores in both people with PD and their caregivers using the EQ-5D VAS were negatively skewed, suggesting that this Australian sample had relatively good HRQoL. The mean EQ-5D VAS score for the participants with PD was 72 (SD = 16) ranging from a low of 15 to the maximum score of 100, with a median of 73 and interquartile range of 25 (60–85). This score was, as expected, lower than the caregivers’ mean of 83 (SD = 13), ranging from 50 to 100, with a median of 85 and interquartile range 16 (77–93); Mann Whitney U: p < 0.001. The results of TTO and VAS Wt components of the EQ-5D essentially reflected the VAS scores. The level of caregiver strain reflected by MCSI scores was similarly somewhat low, with most caregivers scoring less than 16 out of a maximum 26 (median = 5, inter-quartile range = 8, range 0–25). Despite the low median, some caregivers were at near-maximal strain.

### Association between HRQoL in caregiver and care-recipient

**Figure 1:**
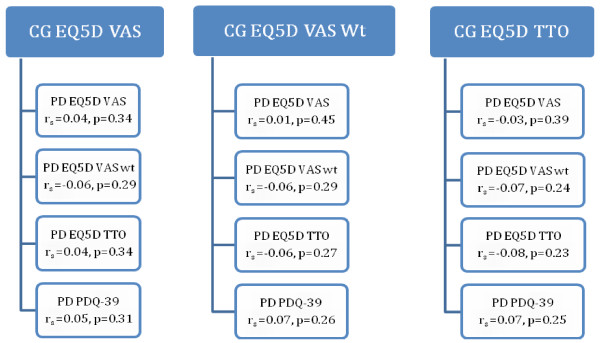
Correlations between HRQoL in care-givers and care-recipients with PD. CG = caregiver, PD = care-recipient with Parkinson’s disease.

As expected, HRQoL scores using the EQ-5D VAS were lower in participants with PD (mean = 72.1, SD = 16.4; range 15–100). Similar distributions were shown using the EQ-5D TTO and VAS Wt methods. No significant correlations were found between HRQoL in caregiver and care-recipient, whether using the generic EQ-5D for PD, or the disease-specific PDQ-39 (See Figure 1). HRQoL in this sample of caregivers and care-recipients was not related.

### Association between caregiver HRQoL and caregiver strain

No significant correlation was found between caregiver strain and caregiver HRQoL irrespective of the measure used (EQ-5D VAS: r_s_ = −0.13, *p* = 0.11; TTO: r_s_ = −0.08, *p* = 0.21; VAS Wt: r_s_ = −0.09, *p* = 0.19). Most caregivers reported relatively low levels of strain (13 or less), coupled with relatively high HRQoL of 75 or more. Of interest, there was no significant correlation between caregiver strain and caregiver age (r_s_ = −0.15, p = 0.079).

### Association between HRQoL in PwPD and caregiver strain

Both the EQ-5D and PDQ-39 were used to evaluate the association between HRQoL in the participants with PD and the level of strain experienced by their caregivers (as indicated by MCSI scores). A small but significant negative relationship was found using the EQ-5D TTO and VAS Wt, but not the unweighted VAS (see Table 2), suggesting that poorer HRQoL in people with PD is associated with higher caregiver strain experienced by their caregivers. This became more obvious when the disease-specific PDQ-39 scores were used. The correlation of PDQ-39 with MCSI scores (r_s_ = 0.3 at *p* < .001) confirmed that poorer self-rated health in people with PD was associated with increased strain in their caregivers.

**Table 2:** Relationship between measures of HRQoL in people with PD and care-giver strain (MCSI)

**Correlated variables**	**Spearman*rho***	**p value**
PD EQ-5D VAS	−0.16	0.061
PD EQ-5D VAS Wt	−0.20	0.025
PD EQ-5D TTO	−0.18	0.042
PD PDQ-39	0.43	0.001

## Discussion

This study is one of the first on HRQoL and caregiver strain in an Australian sample of people caring for family members with PD. By comparison with the literature, both the caregivers and the care-recipients in our sample demonstrated relatively high levels of HRQoL, although that of the people with PD was, as expected, somewhat lower than their caregivers. Previous studies have reported reduced HRQoL in groups of people with PD of similar age and disease duration [29,30] when using the EQ-5D VAS. Others have noted greater emotional distress [31] and increased rates of depression in caregivers of people with PD [5,32], findings that suggest caregiver HRQoL may also be reduced. In the current study, however, the mean EQ-5D VAS scores in our cohort of caregivers were similar to non-caregiving Australians of comparable age [33]. Further, it appeared that caregivers in our sample had higher scores for HRQoL than cohorts in the Netherlands [15,34], Spain [10,35] and the UK [36,37]. However, as the Australian value was within one standard deviation of other results, this difference should be interpreted with caution.

Unlike previous studies [4,38], we failed to find an association between HRQoL in the caregiver and care-recipient. Although Martinez-Martin et al.’s sample was similar to ours in age and mean Hoehn and Yahr status, they used different assessment tools for caregiver HRQoL (the caregiver-specific SQLC: Scale of Quality of Life of Caregivers [39]) and care-recipient HRQoL (EQ-5D [18] and PDQ-8 [40]). The slightly different domains covered potentially impact on attempted comparisons across groups. Differences in culture or facilities between Spain and Australia may also affect the HRQoL of caregiver or of the people with PD. Peters et al. [38] found a moderate correlation in HRQoL between the mental component score of this measure in people with PD and their caregivers in their UK sample of > 700 dyads using the SF12 [41]. Despite using the same instrument for both groups, differences between their cohort and ours may explain why they found a relationship between the two. The Peters et al. cohort was similar in age but may have had more long-standing disease; 35% of patients had been diagnosed for five to ten years and 32% for longer than 10 years, whereas the mean disease duration for our participants was 7.5 years. Another potential difference lies in the fact that the EQ-5D captures information about how people perceive their HRQoL at that time, whereas the SF-12 and PDQ-39 ask about perceptions over the past four weeks. This may lead to under-reporting from the EQ-5D.

Contrary to expectations, no relationship was found between caregiver HRQoL and caregiver strain, perhaps related to the relatively high HRQoL and low levels of strain reported by our cohort. As more than half of our caregivers reported optimal health, the impact of a ceiling effect to the EQ-5D cannot be discounted. An Australian study found a strong tendency of the EQ-5D to allocate an excess of responses a 1.00 value and therefore reduce the capacity of the instrument to discriminate at the upper end of the range [33]. Although MCSI scores were more evenly distributed, some 80% of caregivers reported scores in the lower half of the scale, meaning relatively low levels of strain. At the individual level however, some caregivers reported near-maximal strain, reinforcing the potential of extreme strain in family caregivers.

In comparison with the literature there were no studies found examining the relationship between caregiver strain using the MCSI with caregiver HRQOL. While there have been several which have examined its precursor, the Caregiver Strain Index (CSI), correlations were not reported [37,41]. However several studies have found a statistically significant negative correlation between the Caregiver Burden Index (CBI) and caregiver HRQOL (SQLC) [5,42], whilst another has found a significant negative association with the Zarit Caregiver Burden Inventory (ZCBI) [32].

Our findings of reasonable HRQoL and fairly low caregiver strain may relate to the nature of our sample. Depression is the most commonly cited predictor of reduced HRQoL in people with PD [43] and known to have a negative impact on caregiver HRQoL [44] and stress, strain or burden [31,44]. People with depression, whether patient or caregiver, may be less willing to participate in a research project, especially when the major study required 14 months commitment from participants. Further, dementia was an exclusion criterion (MMSE score of less than 24), potentially contributing to selection bias because cognitive issues in the care-recipient are known to increase caregiver burden [5] and distress [31].

Our group of caregivers was under less strain than has been reported in other papers, perhaps because of relatively higher HRQoL. For example, Peters et al. [38] reported a mean of 11.89 (SD 6.39) (maximum possible for maximal strain is 26 points) on the MCSI, whereas the median for our caregivers was only 5. This may reflect a longer disease duration and considerably poorer HRQoL of their Parkinson’s group (mean PDQ-39 index score of 41 (SD 18), compared to 23 (SD 12) for the participants with PD in our study).

In support of the idea that better HRQoL in care-recipients reduces strain on the caregiver, a significant correlation existed between the HRQoL in the individuals with PD and the level of strain experienced by their caregivers. Greater correlations were found using the disease-specific PDQ-39 than the generic EQ-5D measure. The added strength of the PDQ-39 correlation is not surprising, given that it is disease-specific, comprises 39 detailed items, and evaluates HRQoL over the past four weeks, rather than a single day as in the EQ-5D. Peters et al [38] similarly reported higher correlations between the PDQ-39 and MCSI (r_s_ = 0.56, p < .001) than the generic SF-12 in care-recipients and the MCSI (physical component: r_s_ = −0.33; p < .001; mental component: r_s_ = −0.41, p < .001 [note that SF-12 and MCSI are scored in different directions, leading to negative correlations]).

Understanding the direct impact of patient HRQoL on the levels of caregiver strain is an important result. It has been suggested that families are “the most valuable and also the most vulnerable resource” in the treatment of PD [28]. Recognition of this has led to the development of educational and support programs for family caregivers of people with PD [15,34]. It also suggests that HRQoL should be a routine part of the assessment of patients with PD by health professionals, in conjunction with caregiver strain. Results from the PDQ-39 can inform the treating staff about how important various problems are to the individual, enabling management to be directed towards what is important to the patient, rather than most obvious to the therapist. PDQ-39 items such as difficulties dressing, carrying shopping or getting around the home, can be improved with targeted intervention by physical therapists, occupational therapists or nurses. Prioritising treatment by patient perceptions of difficulty provides emotional as well as physical benefits as the people with PD feels “listened to” and their opinion valued. Improved HRQoL of the care recipients may then flow on to help minimise caregiver strain.

### Limitations

The cross sectional design, appropriate for the aims of the study, meant that issues of causality between variables could not be investigated. A further limitation of this study is that depression was not measured. Depression is a notable factor that contributes to the HRQOL and caregiver strain [9,28,31,32,45] although it was beyond the scope of this study to include measures of depression. Similarly, other pertinent caregiver personal factors that may influence strain and HRQOL such as sense of coherence, coping styles and resilience were not examined. The above factors could be explored in future research on caregiver quality of life and caregiver strain.

This sample cannot be taken to be completely representative of all caregivers. The sample was one of convenience with ambulant clients who had volunteered. It is possible that volunteers with PD and their caregivers who were not overly burdened by their condition could have been more likely to volunteer, or the responses of caregivers may have been in a more positive direction than for the broader community of caregivers of people with PD. Nevertheless, the sampling methods were similar to most other studies which examined the HRQOL of caregivers throughout the world.

## Conclusions

This sample of Australians with PD and their caregivers experienced relatively good HRQoL and there was a generally low level of caregiver strain. However, poorer HRQoL in people with PD was significantly associated with increased strain in caregivers. Assessment of HRQoL may enable targeted interventions that may indirectly reduce caregiver strain.

## Abbreviations

PD, Parkinson’s disease; PwPD, people (or person) with PD; HRQoL, health-related quality of life; MCSI, modified Caregiver Strain Index; PDQ-39, Parkinson’s Disease Questionnaire −39 items; EQ-5D, Euroquol 5D; 6MWT, timed 6 metre Walk Test; RCT, randomized controlled trial; VAS, Visual Analogue Scale; VAS Wt, weighted Visual Analogue Scale; TTO, Time trade-off method; SQLC, Scale of Quality of Life of Caregivers; SF12, Short Form 12.

## Competing interests

The authors declare that they have no competing interest.

## Authors’ contributions

DK, MM, JM, FH, RI, HM, JW and AM conceived the idea for the study and participated in the study design. MD, JM, MM and DK conducted participant recruitment, data collection, data analysis and project management. DK, FH, MM, JM, and BA drafted the initial manuscript for submission. All authors have reviewed drafts of the manuscript and have provided final approval of the version to be published.

## Pre-publication history

The pre-publication history for this paper can be accessed here:

http://www.biomedcentral.com/1471-2377/12/57/prepub
